# ‘Druggable’ alterations detected by Ion Torrent in metastatic colorectal cancer patients

**DOI:** 10.3892/ol.2014.2047

**Published:** 2014-04-08

**Authors:** WEIJIA FANG, MILAN RADOVICH, YULONG ZHENG, CAI-YUN FU, PENG ZHAO, CHENGYU MAO, YI ZHENG, SHUSEN ZHENG

**Affiliations:** 1 Department of Hepatobiliary and Pancreatic Surgery, The First Affiliated Hospital, School of Medicine, Zhejiang University, Hangzhou, Zhejiang 310006, P.R. China; 2 Department of Surgery, Indiana University School of Medicine, Indianapolis, IN 46202, USA; 3 Laboratory of Proteomics and Molecular Enzymology, School of Life Sciences, Zhejiang Sci-Tech University, Hangzhou, Zhejiang 310018, P.R. China

**Keywords:** druggable alterations, Ion Torrrent, metastasic colorectal cancer, formalin-fixed paraffin-embedded

## Abstract

The frequency and poor prognosis of patients with metastatic colorectal cancer (mCRC) emphasizes the requirement for improved biomarkers for use in the treatment and prognosis of mCRC. In the present study, somatic variants in exonic regions of key cancer genes were identified in mCRC patients. Formalin-fixed, paraffin-embedded tissues obtained by biopsy of the metastases of mCRC patients were collected, and the DNA was extracted and sequenced using the Ion Torrent Personal Genome Machine. For the targeted amplification of known cancer genes, the Ion AmpliSeq™ Cancer Panel, which is designed to detect 739 Catalogue of Somatic Mutations in Cancer (COSMIC) mutations in 604 loci from 46 oncogenes and tumor suppressor genes using as little as 10 ng of input DNA, was used. The sequencing results were then analyzed using the Ampliseq™ Variant Caller plug-in within the Ion Torrent Suite software. In addition, Ingenuity Pathway software was used to perform a pathway analysis. The Cox regression analysis was also conducted to investigate the potential correlation between alteration numbers and clinical factors, including response rate, disease-free survival and overall survival. Among 10 specimens, 65 genetic alterations were identified in 24 genes following the exclusion of germline mutations using the SNP database, whereby 41% of the alterations were also present in the COSMIC database. No clinical factors were found to significantly correlate with the alteration numbers in the patients by statistical analysis. However, pathway analysis identified ‘colorectal cancer metastasis signaling’ as the most commonly mutated canonical pathway. This analysis further revealed mutated genes in the Wnt, phosphoinositide 3-kinase (PI3K)/AKT and transforming growth factor (TGF)-β/SMAD signaling pathways. Notably, 11 genes, including the expected APC, BRAF, KRAS, PIK3CA and TP53 genes, were mutated in at least two samples. Notably, 90% (9/10) of mCRC patients harbored at least one ‘druggable’ alteration (range, 1–6 alterations) that has been linked to a clinical treatment option or is currently being investigated in clinical trials of novel targeted therapies. These results indicated that DNA sequencing of key oncogenes and tumor suppressors enables the identification of ‘druggable’ alterations for individual colorectal cancer patients.

## Introduction

In 2013, colorectal cancer had the third highest incidence of new cases and the third highest rate of cancer mortality in the USA, with 142,280 and 50,830 individuals, respectively (1). The occurrence and development of this lethal disease is a multi-step process involving multiple gene mutations. The diversity and complexity of somatic mutational processes that underlie carcinogenesis in humans is being revealed through mutational patterns hidden within cancer genomes (2). A variety of genomic consortia, including The Cancer Genome Atlas (TCGA) and the International Cancer Genome Consortium, are attempting to catalog all somatic mutations occurring in major cancer types. In addition, the Catalogue Of Somatic Mutations In Cancer (COSMIC) has served as a central repository designed to store and exhibit somatic mutation information derived from the cancer genome consortia and from the literature (3–5). Driving the massive data collection is the use of next-generation sequencing (NGS), which has the ability to probe millions of DNA fragments for mutations and is subsequently enabling clinicians to more accurately gauge the risk of developing cancer and tailor therapies to treat cancers with specific genetic mutations (6). The Ion Torrent Personal Genome Machine (PGM; Invitrogen Life Technologies, Carlsbad, CA, USA) presents an emerging NGS approach that relies on non-optical semiconductor sequencing technology with a rapid turnaround time (7). The deep coverage achieved by the PGM makes it possible to detect somatic mutations in tumor cells with low allele frequency, which may not be detected by conventional Sanger sequencing.

Notably, much of the data reported thus far by the Cancer Genomic Consortium using NGS has focused on sequencing from the primary tumor, with limited data on ‘druggable’ mutations present in the metastases. The current study used this emerging technology to detect somatic mutations in formalin-fixed, paraffin-embedded (FFPE) tissues obtained from the metastatic nodules of metastatic colorectal cancer (mCRC) patients, in order to identify somatic alterations suitable for anticancer drug treatment.

## Patients and methods

### Patients

FFPE tissues obtained from patients with mCRC were collected from The First Affiliated Hospital, School of Medicine, Zhejiang University (Hangzhou, China). All patients provided written informed consent and the study protocol was approved by the Institutional Ethics Committee of The First Affiliated Hospital. Patient information, including age, gender, diagnosis, positive lymph node number, response rate, disease-free survival following primary surgery, overall survival following salvage chemotherapy, number of metastasic organs and chemotherapy regimen were recorded. Chemotherapy efficacy evaluation was performed according to the Response Evaluation Criteria in Solid Tumors guidelines, version 1.1 (8).

### NGS sequencing

DNA preparation was performed, as described previously (9). The DNA was then sequenced using the PGM (Invitrogen Life Technologies) according to the manufacturer’s instructions. For the targeted amplification of known cancer genes, the Ion AmpliSeq™ Cancer Panel (Invitrogen Life Technologies), which is designed to detect 739 COSMIC mutations in 604 loci from 46 oncogenes and tumor suppressor genes using as little as 10 ng of input DNA, was used. Next, a template was prepared using the Ion PGM 200 Xpress template kit (Invitrogen Life Technologies) and sequencing was performed using the Ion Sequencing kit version 2.0 on an Ion 316 chip. Data were analyzed using the Ampliseq™ Variant Caller plug-in within the Ion Torrent Suite software (Invitrogen Life Technologies). The sequences of all primers and probes are available on request.

### Validation Sanger sequencing of KRAS and FGFR3

The sequencing template used for KRAS was a 170-bp polymerase chain reaction (PCR) fragment of the KRAS gene, generated using the following primers: Forward, 5′-AAGGCCTGCTGAAAATGACTG-3′ and reverse, 5′-AGAATGGTCCTGCACCAGTAA-3′ [Generay Biotech (Shanghai) Co., Ltd., Shanghai, China]. The PCR conditions used were as follows: 40 cycles of predenaturation for five min at 95°C, denaturation at 95°C for 20 sec, annealing at 60°C for 20 sec and elongation at 72°C for 20 sec, followed by a final extension at 72°C for five min.

The sequencing template used for FGFR3 was a 296-bp PCR fragment of the FGFR3 gene, generated using the following primers: Forward, 5′-GTGTGTATGCAGGCATCCTCAGC-3′ and reverse, 5′-ATGGTGAGCAGAGACGAGGAGAGG-3′ [Generay Biotech (Shanghai) Co., Ltd.]. The PCR conditions used were as follows: 40 cycles of predenaturation for five min at 95°C, denaturation at 95°C for 20 sec, annealing at 62°C for 20 sec and elongation at 72°C for 20 sec, followed by a final extension at 72°C for five min.

The PCR products were then purified using the shrimp alkaline phosphatase/exonuclease PCR clean method [New England Biolabs (UK) Ltd., Hitchin, UK]. Next, the purified samples (2 μl) were used directly for a sequencing reaction using the Big Dye Terminator cycle sequencing mix, version 3.1 (Applied Biosystems, Carlsbad, CA, USA). Sequencing reactions were then performed for the two DNA strands using the PCR oligonucleotides (3.2 pmol) as respective primers. Dye purification was performed using alcohol/sodium acetate precipitation, and subsequent sequence analysis was conducted using an ABI 3130 genetic analyzer (Applied Biosystems).

### Gene pathway analysis

Ingenuity pathway analysis (IPA; Qiagen, Valencia, CA, USA) was used for core analysis to identify the existing metastasis network.

### Statistical analysis

Statistical analyses were conducted using SPSS version 20.0 (IBM, Armonk, NY, USA). All tests of significance were two-sided and P≤0.05 was considered to indicate a statistically significant difference. Cox regression analysis was used to investigate a potential correlation between the alteration numbers and clinical factors, including age, gender, diagnosis, positive lymph node number, response rate, disease-free survival following primary surgery, overall survival following salvage chemotherapy, number of metastasic organs and chemotherapy regimen.

## Results

### Patient characteristics

A total of 10 mCRC patients were enrolled in the current study between April 2007 and August 2010 (Table I). The median age was 60 years (range, 37–73 years) and the patients consisted of five males and five females.

### Overall gene alterations

**Figure 1:**
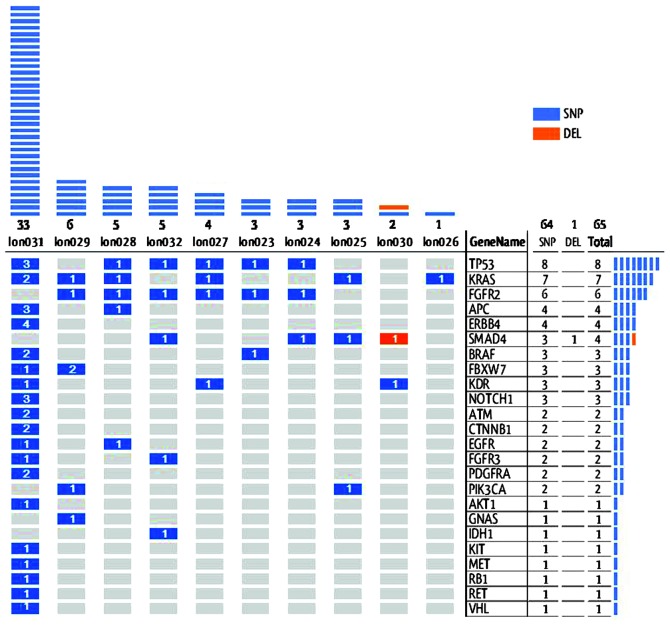
Overall gene alteration patterns for 24 target genes in 10 samples. The blue bars represent SNPs whereas the red bars represent DELs. The numbers inside the bars show the alteration number and the upper columns show the number of alterations in each sample. Sample 31 exhibited the maximum number of 33 alterations, whereas sample 1 only exhibited one alteration. The right columns show the number of alterations in each gene; TP53 exhibited the maximum number of eight alterations, whereas eight genes, including AKT1, GNAS and IDH1, only exhibited one. SNPs, single nucelotide polymorphisms, DEL, deletion; PI3K, phosphoinositide 3-kinase.

Among the 10 specimens, 65 genetic alterations were identified in 24 genes, following the exclusion of germline mutations according the single nucleotide polymorphism (SNP) database, as shown in Fig. 1.

Among these alterations, 41% were present in the COSMIC database. These alterations confirmed by COSMIC were all SNPs, divided into missense (83%) and nonsense (17%) changes. Notably, four genes exhibited >1 alteration; APC (n=2), FBXW7 (n=2), TP53 (n=3) and KRAS (n=5). No clinical factors were found to significantly correlate with the alteration numbers in patients by statistical analysis.

### Sanger sequencing validation

Sanger sequencing of KRAS revealed that all results were consistent with PGM, with the exception of one. Sample 31 was wild-type, however, PGM identified the G13D mutation (c.38G>A; COSMIC 532). PGM also revealed that the percentage of mutations at the cell level of this sample was 26.6%. Sanger sequencing of FGFR3 revealed that all samples were wild-type with seven consecutive ‘C’ repeats (GCCTGCGCAGCCCCCCCAAGAAA). However, PGM revealed a ‘C’ to ‘G’ change in eight samples, with the sequence ‘GCCTGCGCAGGCCCCCCAAGAAA.

### Pathway analysis of detected somatic mutations

**Figure 2:**
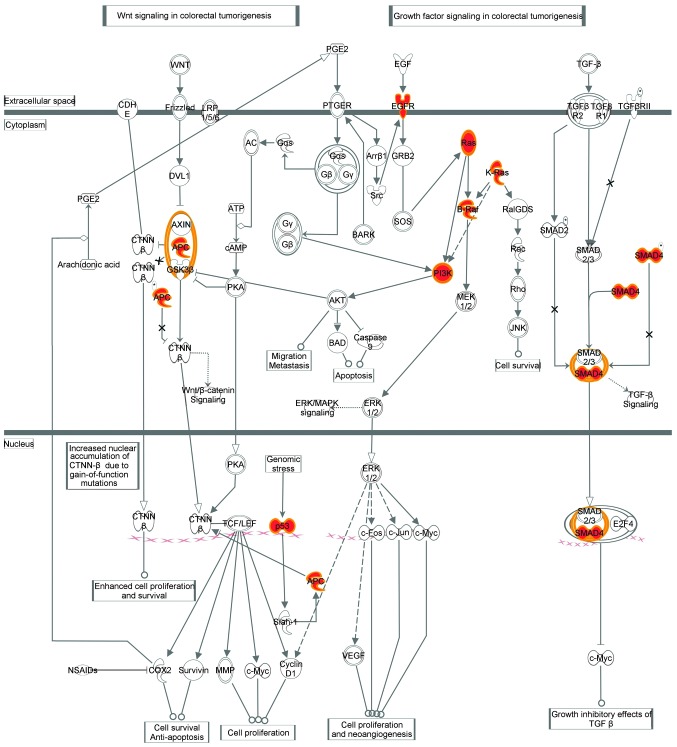
Colorectal cancer metastasis signaling pattern. Colorectal cancer metastasis signaling is the the most commonly mutated canonical pathway, and includes Wnt, phosphoinositide 3-kinase (PI3K)/AKT and transforming growth factor (TGF)-β/SMAD signaling. The key mutated genes detected in the present study are highlighted in red.

The IPA identified ‘colorectal cancer metastasis signaling’ as the most commonly mutated canonical pathway, which includes Wnt, phosphoinositide 3-kinase (PI3K)/AKT and transforming growth factor (TGF)-β/SMAD signaling (Fig. 2).

**Figure 3:**
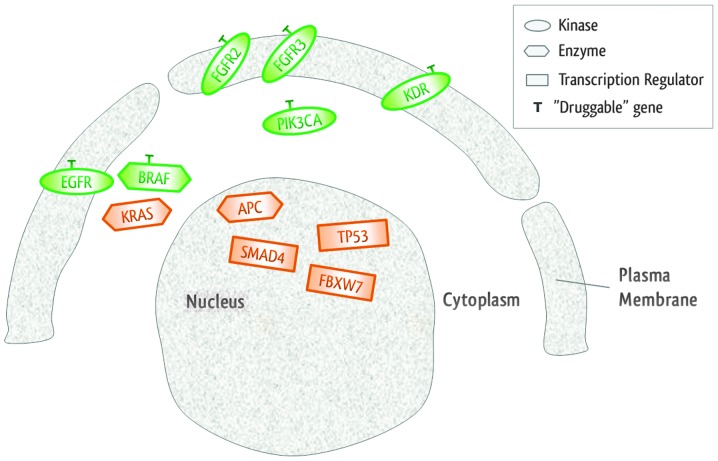
Novel mutated genes and their locations at cell level. The ‘druggable’ genes detected in the present study are highlighted in green.

Notably, 90% (9/10) patients harbored at least one ‘druggable’ alteration (range, 1–6 alterations) that has been associated with a clinical treatment option or is currently being investigated in clinical trials of novel targeted therapies, as shown in Table II. In addition, IPA clarified that there were six ‘druggable’ genes with specific target drugs, and 90% of samples exhibited at least one of them, as shown in Fig. 3.

## Discussion

Oncology is progressing away from organ-of-origin-based management strategies towards the more refined strategy of using targeted therapies driven by a tumor’s molecular characteristics. At present, NGS aids in ‘building bridges’ between molecular screening and clinical reality, with an accuracy of 96.1% in comparison to Sanger sequencing (10). Furthermore, PGM is aiming to become a ‘point-of-care’ NGS platform (11). In the present study, PGM identified 65 genetic alterations in 24 genes using as little as 10 ng of input DNA of each sample. In addition, PCM has the ability to reveal alterations at very low allele frequencies, a major limitation of conventional Sanger sequencing. In patient 31, as Sanger sequencing revealed a ‘wide-type’ result prior to chemotherapy, cetuximab was administered to the patients, which achieved a good response. However, the PGM also revealed a KRAS G13D mutation, presenting an noteworthy situation, as a randomized clinical trial previously demonstrated that tumors with a KRAS G13D mutation may also be sensitive to cetuximab in colorectal cancer patients (12).

A limitation of the PGM is the large number of false-positive indels, which result from homopolymer errors. To overcome this, the current study used the Ampliseq™ Variant Caller plug-in within the Ion Torrent Suite software, which found only one deletion alteration among the 65 somatic variants detected. In addition, due to the lack of matched germline samples, the SNP database was used to exclude germline variants prior to the generation of the final results, However, difficulties remain in differentiating somatic mutations from rare germline inherited SNPs. An unexpected false-positive indel in FGFR3 was also observed, which was possibly due to its seven consecutive identical base ‘C’ repeats, which are prone to errors in PGM analysis.

To obtain a pathway-centric analysis of the data, the current study used IPA, which combines advanced pathway enrichment analysis with the pathway topological analysis to aid in the identification of the most relevant metabolic pathways involved in diseases and cellular processes (13). IPA identified significant cancer pathways, including those of Wnt signaling, PI3K/AKT and TGF-β/SMAD, which are known to be frequently activated in cancer. In addition, mutations in the APC gene have been identified as the initiating event in the inherited and spontaneous forms of mCRC. Furthermore, when this tumor suppressor gene is mutated, its ability to regulate the Wnt pathway is lost (14). The past decade has revealed that the PI3K/AKT signaling pathway is among the most highly mutated pathways in human carcinogenesis (15). Mutations in this pathway occur in the majority of cancers contributing to the resistance to apoptosis, the deregulation of proliferation and changes in the metabolism characteristics of transformed cells (16). Finally, the function of members of the TGF-β family is exerted via specific kinase receptors and intracellular SMAD transcription factors, including the common mediator Smad4. The initiation of adenocarcinomas of the gastrointestinal tract and squamous cell carcinomas of the skin can involve the loss of SMAD4 (17).

Notably, 11 genes, including the expected APC, BRAF, KRAS, PIK3CA and TP53 genes, were mutated in at least two samples of the current study, which is similar to the gene list revealed by the TCGA Network in 2012 (18).

This phenomenon indicates that almost all colorectal cancer patients may have the chance to be treated with at least one target drug according to their ‘druggable’ genes. For example, in the present study, vemurafenib was considered to target patients with the BRAF V600E mutation (19). In addition, the anti-epidermal growth factor receptor, cetuximab, has been proven to be of great success in mCRC treatment (20). Other examples of drug and targeted gene pairs are as follows: Palifermin and FGFR2, pazopanib and FGFR3, AEE 788 and KDR, and BEZ235 and PIK3CA (21–23).

The majority of somatic mutations in this tumor class have great potential to provide a variety of target drugs for cancer patients. However, challenges remain in translating sequencing information into clinical practice. Therefore, identification of genetic factors that affect the response to treatment are essential to identify and develop next generation medicines that target the ‘druggable’ alterations of patients.

## Figures and Tables

**Table I:** Patient characteristics and clinical outcome.

Patient number	Gender	Age, years	Primary location	Positive lymph node	Response rate (first-line chemotherapy)	Disease-free survival, days	Overall survival, days
23	Male	60	Rectum	4	PR	1125	449
24	Female	64	Colon	2	SD	594	Alive
25	Male	60	Rectum	4	PD	403	126
26	Female	59	Rectum	/	SD	518	Alive
27	Male	73	Colon	0	PD	1212	74
28	Female	51	Rectum	0	PD	814	64
29	Female	69	Rectum	2	PD	1096	Alive
30	Male	68	Colon	/	PR	/	852
31	Male	37	Colon	/	PR	/	420
32	Female	57	Rectum	/	PR	/	Alive

The use of ‘/’ refers to patients who had metastatic disease at first diagnosis and therefore surgery could not be performed, so the information regarding lymph nodes is absent. ‘Disease-free survival’ refers to the days between primary surgery and relapse, and ‘overall survival’ refers to the days between the salvage chemotherapy and disease progression. PR, partial response; SD, stable disease; PD, progressive disease.

**Table II:** Ingenuity pathway analysis identified genes and corresponding drugs.

Gene	Location	Type	Drug(s)
APC	Nucleus	Enzyme	
BRAF	Cytoplasm	Enzyme	Vemurafenib, sorafenib
EGFR	Plasma membrane	Kinase	Cetuximab, panitumumab and BMS-599626, among others
FBXW7	Nucleus	Transcription regulator	
FGFR2	Plasma membrane	Kinase	Palifermin
FGFR3	Plasma membrane	Kinase	Pazopanib
KDR	Plasma membrane	Kinase	Sunitinib, axitinib and AEE 788, among others
KRAS	Cytoplasm	Enzyme	
PIK3CA	Cytoplasm	Kinase	SF-1126, PX-866 and NVP-BEZ235, among others
SMAD4	Nucleus	Transcription regulator	
TP53	Nucleus	Transcription regulator	
